# Genetic and Biochemical Alterations in Non-Small Cell Lung Cancer

**DOI:** 10.1155/2012/940405

**Published:** 2012-08-15

**Authors:** Jackie L. Johnson, Smitha Pillai, Srikumar P. Chellappan

**Affiliations:** ^1^Cancer Biology Ph.D. Program, University of South Florida, Tampa, FL 33612, USA; ^2^Department of Tumor Biology, H. Lee Moffitt Cancer Center and Research Institute, 12902 Magnolia Drive, Tampa, FL 33612, USA

## Abstract

Despite significant advances in the detection and treatment of lung cancer, it causes the highest number of cancer-related mortality. Recent advances in the detection of genetic alterations in patient samples along with physiologically relevant animal models has yielded a new understanding of the molecular etiology of lung cancer. This has facilitated the development of potent and specific targeted therapies, based on the genetic and biochemical alterations present in the tumor, especially non-small-cell lung cancer (NSCLC). It is now clear that heterogeneous cell signaling pathways are disrupted to promote NSCLC, including mutations in critical growth regulatory proteins (K-Ras, EGFR, B-RAF, MEK-1, HER2, MET, EML-4-ALK, KIF5B-RET, and NKX2.1) and inactivation of growth inhibitory pathways (TP53, PTEN, p16, and LKB-1). How these pathways differ between smokers and non-smokers is also important for clinical treatment strategies and development of targeted therapies. This paper describes these molecular targets in NSCLC, and describes the biological significance of each mutation and their potential to act as a therapeutic target.

## 1. Introduction

 Lung cancer is the leading cause of cancer-related mortality, annually resulting in more than one million deaths worldwide. In the United States itself, there would have been 222,000 new cases of lung cancer diagnosed in 2010, with about 157,000 deaths [1]. Death from cancers of the lung and the respiratory system would exceed the number of deaths from cancers of breast, colon, pancreas, and the prostate combined. Lung cancer is the leading cancer site in males, comprising 17% of the total new cancer cases and 23% of the total cancer deaths worldwide [1, 2]. Non-small cell lung cancer (NSCLC) accounts for about 80% of all lung cancer cases and is strongly correlated with smoking habits. Small cell lung cancer is almost exclusively diagnosed in smokers, with about 90% of the patients being smokers or former smokers [3]. Despite the strong linkages between smoking and lung cancer, approximately 30% of smokers with lung cancer continue to smoke following their diagnosis [4]. Further, as patients recover from treatment, adapt to a cancer diagnosis, and receive less frequent followup, smoking relapse may become more pronounced [5]. 

 Although smoking is the major risk factor for lung cancer, about 25% of lung cancers occur in never smokers [3] and NSCLC in nonsmokers causes more mortality worldwide than pancreatic and prostate cancers combined [3, 6]. This combined with the fact that only 10–20% of smokers are affected by NSCLC suggest that genetic susceptibility and environmental factors also contribute to the risk of NSCLC. Studies in the past decade have identified different molecular signatures associated with lung cancer in smokers and never smokers; these include differential expression of genes as well as mutations in different genes [3, 7, 8]. The etiology of lung cancer in smokers and nonsmokers is also different, with women comprising a larger proportion of lung cancer among nonsmokers [9, 10]. The histology and location of cancer also show differences in smokers and nonsmokers, with adenocarcinoma being the most prevalent histology in nonsmokers; both adenocarcinomas and squamous-cell carcinomas are widespread in smokers. In addition, the entire spectrum of nonsmall cell histological subtypes can be found in lung cancers from smokers [11, 12]. 

 At the molecular level, non-small cell lung cancer in never smokers are more likely to have mutations in epidermal growth factor receptor (EGFR) tyrosine kinase and patients harboring EGFR mutations show good response to its inhibitors compared to patients with tobacco-associated lung cancer [13, 14]. Mutations in KRAS and TP53 are more common among lung cancer in smokers, along with alterations in additional growth promoting pathways [15]. Treatment options vary for NSCLC in smokers and nonsmokers, and it can be imagined that further characterization of genetic alterations in NSCLC will lead to the development of novel therapeutic options to treat this disease. To this end, major discoveries from next generation sequence analyses have provided a high-resolution glimpse into the complexities of NSCLC genomes. Clinically detectable lung tumors have been shown to harbor frequent genetic and epigenetic aberrations (>20 per tumor) [16]. Such analysis has identified gene fusions including echinoderm microtubule-associated protein-like 4-anaplastic lymphoma kinase (EML4-ALK) [17–20] and more recently in the kinesin family 5B (KIF5B-Ret) proto-oncogene [21–23]. These fusions represent novel drivers of NSCLC, and exciting new therapeutic targets. This paper highlights the most common genetic and molecular alterations in NSCLC in addition to newly identified lung cancer mutations. 

## 2. Activation of Growth-Promoting Signaling Pathways

### 2.1. K-RAS

 Lung tumors in humans are characterized by their histological types and are assigned as either small-cell lung cancers or non-small-cell lung cancers (NSCLC) [24]. Accounting for nearly 87% of total lung cancers, NSCLC are further distinguished into three subtypes: squamous-cell carcinoma, large-cell carcinoma, and adenocarcinoma, where adenocarcinoma has the highest clinical presentation, accounting for nearly 50% of lung cancers diagnosed [25]. In 30% of adenocarcinomas, mutation of the KRAS proto-oncogene is the driving force behind oncogenic transformation, and similar mutations are found to a lesser extent (about 5%) in the squamous-cell carcinoma subtype [25]. In addition, mutation of KRAS is more prevalent in patients who are current or former smokers (25%) than never smokers (6%) [26].

 The RAS family was originally identified, like many other oncogenes, by studies conducted on cancer-initiating retroviruses. The Harvey (HMSV) and Kirsten (KMSV) murine sarcoma RNA tumor viruses, named HRAS and KRAS after their respective discoverers, were shown to induce sarcoma and erythroleukemia in rats in the 1960s [27–29]. In the early 1980s, similar genes were identified by several groups, who isolated the human ortholog of these transforming genes from various human cancer cell lines [30–34]. Another RAS family member was identified from a human neuroblastoma cell line, neuroblastoma RAS (NRAS), and is also mutated in various human cancers [35, 36]. Since the discovery of these prominent RAS oncogenes, nearly 150 human family members in the RAS superfamily have been identified with evolutionarily conserved orthologs in *Drosophila, S. cerevisiae, C. Elegans, S. pombe,* and plants [37, 38]. 

 The three human RAS genes encode four highly homologous proteins, where *KRAS4A* and *KRAS4B *result from alternate splicing mechanisms, and differ only in their 25 C-terminal residues [39, 40]. Functionally, Ras proteins are guanosine diphosphate (GDP) and guanosine triphosphate (GTP) regulated switches, whereas in a normal quiescent cell, Ras is GDP bound, and hence inactive [41]. Upon growth factor engagement to receptors at the cell surface, guanine nucleotide exchange factors (e.g., Son of Sevenless, SoS) stimulate the formation of Ras-GTP [29]. This form of Ras can then bind to a plethora of downstream effector targets, including well-studied Raf kinases and mitogen-activated protein kinases (MAPK) and phosphoinositide 3-kinases (PI3 K), and transmit these extracellular cues to regulate cell growth, motility, differentiation, senescence, or even cell death [42]. After the signal is transmitted, Ras-GAPs, or GTPase activating proteins (e.g., NF1, neurofibromin), catalyze GTP hydrolysis and the formation back to the inactive form, Ras-GDP [41, 43]. In addition to regulation by Ras-GEFs and Ras-GAPs, Ras proteins are tethered to the plasma membrane by farnesyl moities that are posttranslationally added in their c-termini by Farnesyltransferases [44]. This association with the plasma membrane through farnesyl modification is crucial for eliciting downstream signals, and has therefore been exploited as an effective drug target for sequestering Ras-mediated signaling *in vitro, in vivo, *and more recently in clinical trials [45–48]. 

 Ras mutations found in human cancers generate mutated proteins which have single amino acid substitutions at codon G12, G13, or Q61 [49, 50]. These mutations render Ras proteins GDP insensitive, which leads to constitutive activation of downstream effectors [51]. In lung cancer specifically, mutations are found in G12 and G13, but whether these mutations correlate with disparities in prognosis, metastasis, and survival is unclear [52, 53]. Since no available drugs block KRAS directly, efforts have been made to evaluate other potential targets of the RAS pathway that function downstream [54]. To this end, the weak RAF inhibitor, Sorafenib, was used in the BATTLE trial with modest efficacy, with the no progression rate at eight weeks being 46% [55]. 

 To characterize a phenotype for somatic KRAS gene mutations *in vivo*, Tyler Jacks' lab created a murine model of spontaneous onset lung cancer by utilizing a variation of “hit and run” gene targeting of the mutant Ras allele commonly found in humans, G12D [56]. In this model, 100% of mice developed multifocal lung nodules, and had a median survival of 200 days compared to over 800 days for wild-type control littermates [56]. In the same study, mice harboring nullizygous mutation for the tumor suppressor TP53 in the G12D background developed more aggressive lung tumors resulting in further reduction in mean survival, in addition to a broad spectrum of tumors in other organs [56]. 

 Further studies determined an NF-*κ*B-dependent mechanism that caused aggressive tumor formation in these RasG12D mutant, TP53 nullizygous mouse models of lung adenocarcinoma [57]. When cell lines were derived from these tumors, NF-*κ*B p65 DNA binding activity was significantly higher in mice with mutant TP53 when compared to wild-type controls. In addition, nuclear p65 was higher in the TP53 mutant cells both *in vitro* and *in vivo*. Interestingly, knockdown of p65, but not the related protein c-Rel, led to reduced cell viability, cleavage of caspase-3, and induction of apoptosis, demonstrating that the p65-dependent NF-*κ*B signaling pathways are required for survival of cell lines derived from these mouse models of NSCLC. These data compliment the observation that NF-*κ*B signaling is important for chemically induced models of lung cancer as well [58].

 One strategy for targeting KRAS-driven lung cancer is to determine crucial downstream signaling cascades that, when inhibited, cause cell death in the presence of the driver mutation, but not the presence of a wild-type allele. In this vein, meta-analysis of RNAi screens have collectively identified through “Hairpin analysis” and RNAi gene enrichment ranking (RIGER) 45 possible KRAS synthetic lethal interactions, with TBK1 being the most significant [59]. Interestingly, TBK1 is a noncanonical I*κ*B kinase that activates NF-*κ*B antiapoptotic signals involving c-Rel and BCL-XL to promote cell survival. Inhibiting TBK1 induces apoptosis exclusively in cell lines that require KRAS [59]. 

 Using the same KRasG12D mouse model, the Barbacid lab has validated another synthetic lethal interaction between RasG12D mutation and cyclin-dependent kinase-4 (CDK4) ablation, demonstrating the requirement for nonredundant, interphase CDK4 in triggering oncogenesis in a RasG12D mutant mouse [60]. CDK4 ablation caused an immediate senescence response in the lungs of RasG12D animals, though not with CDK2 or CDK6. Further, in advanced stage tumors, *cre*-mediated ablation of CDK4 induced senescence as well, suggesting that targeting CDK4 in already developed tumors could be an effective therapeutic strategy. When a selective CDK2 and CDK4 inhibitor, PD0332991, was tested in mice with already established tumors detected by CT there was a significant decrease from 25-fold in the vehicle treated mice to 6-fold in the PD0332991-treated group [60]; however there was no onset of senescence, and tumor burden did not regress, but rather increased minimally. These results suggest that induction of a senescence response must require a strong, prolonged inhibition of Cdk4 activity, which was probably not achieved with the PD0332991 inhibitor. Proving that senescence could not be used as a marker for clinical efficacy of this inhibitor, this study gives application to the development of novel, more robust CDK4 inhibitors. 

 One of the most prevalent pathways affected by oncogenic mutations in cancers is the RAS/RAF/MEK/ERK signaling cascade, and NSCLC is no exception [61]. Although perturbation can occur at multiple nodes as a result of an initial KRasG12D mutation, recent studies have elegantly illustrated how each individual member of this cascade is crucial for the onset of NSCLC [62]. Firstly, although single elimination of ERK1 or ERK2 has no effect on survival, simultaneously deleting both alleles increased survival by 40%. Similar results were observed upon single deletion of either Mek1 or Mek2, where both are dispensable for tumor development, but combined deletion of both results in nearly 100% increase in survival. This calls into question whether the 2 out of 207 primary lung tumors with single-somatic activating-point mutations in MEK1 were merely correlative, rather than causative events, or whether the animal model of NSCLC is an accurate representation of the human disease [63]. 

 It has been shown that the retinoblastoma tumor suppressor gene, *RB*, itself is rarely mutated in NSCLC [64, 65], but is widely altered in SCLC [66]. At the same time, Rb protein is inactivated in a high percentage of NSCLC through the inactivation of the p16INK4 gene, which results in elevated cyclin dependent kinase activity, as described in a later section. It is well established that phosphorylation of the Rb protein by cdks associated with D- and E-type cyclins leads to its inactivation, facilitating S-phase entry and cell-cycle progression [67]. Studies from our lab had shown a more direct link between the Ras-Raf-MAP kinase cascade and Rb inactivation. Our studies had shown that the kinase C-Raf (Raf-1) physically interacts with Rb early in the cell cycle, facilitating its complete inactivation by cyclin-dependent kinases [68, 69]. Interestingly, the amount of Raf-1 associated with Rb was elevated in NSCLC tumors compared to adjacent normal tissue [70], suggesting that the enhanced interaction of C-Raf with Rb might have contributed to oncogenic process. Further, disruption of the Rb-Raf-1 interaction using an eight-amino-acid peptide [69] or a small molecule disruptor [71] inhibited the growth of NSCLC tumors in xenograft models, suggesting that disrupting the Rb-Raf-1 interaction might be a viable strategy to combat NSCLC, especially those harboring K-Ras mutations [72, 73]. The necessity of inactivating Rb for K-Ras to initiate NSCLC was further demonstrated in elegant mouse models from the Sage lab [74]. 

 Finally, the loss of B-RAF had no effect on tumorigenesis, where pERK levels remained unchanged despite the mutation, however loss of C-RAF resulted in an 83% increase in survival. This increase was a consequence of a reduced number of tumors [62]. Taken together, these studies highlight two main pathways working to promote tumorigenesis of KRAS-driven lung tumors in mice: the NF*κ*B pathway and the MAPK cascade. Whether these pathways are equally critical to human tumor initiation and progression remains less clear.

### 2.2. EGFR

Whereas normal cells utilize stringent regulatory programs for receptor tyrosine kinase (RTK) functions, mutation and deregulated expression of RTKs is a common event in many cancer subtypes, including NSCLC. The epidermal growth factor receptor (EGFR) is a member of the ERBB receptor family, and is composed of a ligand binding domain on the extracellular surface and an intracellular domain that contains the tyrosine kinase motif. EGFR can be activated by a variety of extracellular cues, including epidermal growth factor, TGF-*α*, and Amphiregulin [75]. Once ligand binding is engaged, the formation of homo- and heterodimers occurs, resulting in transphosphorylation and activation of the receptors. The phosphorylation of these receptors creates a prime docking site for intracellular adaptor proteins and kinases to elicit further downstream signals.

EGFR deregulation is common in a variety of tumor subtypes, including NSCLC, where protein overexpression is observed in up to 62% [76–78]. In addition to protein overexpression, EGFR is commonly somatically mutated in close to 40% of adenocarcinomas and 30% of adenosquamous NSCLC (mutations occurring ~50% of nonsmokers and 5–15% smokers) [79–81]. Kinase domain mutations are generally *activating mutations* leading to a ligand-independent activation of tyrosine kinase (TK) activity. The activating mutations of the EGFR gene are found in the first four exons (18–21) of the TK domain [78, 82, 83]. These mutations are classified into three classes, with majority of EGFR-TKI sensitizing mutations falling into class 1 and 2. Class 1 mutations are in frame deletions in exon 19 and account for about 44% of all EGFR TK mutations. Class 2 mutations are single nucleotide substitutions that result in amino acid alteration. Most predominant in this class of mutation is in exon 21, which substitutes an arginine for a leucine at codon 858 (L858R), and this mutation accounts for about 41% of all EGFR TK-activating mutations [82]. Class 3 mutations are in frame duplications or insertions in exon 20 and account for 5% of all EGFR TK-activating mutations. 

 In addition to the above mutations, deletions in exon 19 and L858R mutations constitute 90% of all EGFR-activating mutations and are termed *classical *activating mutations [78]. Classical EGFR mutations occur preferentially in specific subsets, such as patients with adenocarcinoma histology, never smokers, those with East Asian ethnicity, and female patients. In a recent study by Shigematsu et al., 45% of never smokers had EGFR mutations, whereas only 7% of smokers had EGFR mutation [84]. The high frequency of EGFR mutations in never smokers is consistent across different ethnic and geographic groups. 

 Since EGFR is one of the most frequently deregulated genes in NSCLC, it became one of the first rationally selected molecules for targeted therapy. Initial efforts were used to block the ligand-receptor interaction with monoclonal antibodies, however new small molecules that target the TK activity of EGFR (gefitinib and erlotinib) have had remarkable efficacy in NSCLC patients with mutations in the EGFR gene [77, 85]. Unfortunately, lung cancers with drug sensitive EGFR mutations that initially respond to gefitinib or erlotinib eventually develop acquired resistance from between six months to two years later [86]. Approximately 50% of NSCLC patients who respond initially to reversible first generation EGFR TKIs, eventually develop resistance by aquiring a second recurrent missense mutation in the EGFR kinase domain. The most common (>90%) mutation involves a substitution of methionine for threonine at position 790 (T790 M) in exon 20 [87, 88]. The bulkier methionine residue at position 790 sterically hinders the interaction with inhibitor, effectively preventing binding to the EGFR kinase domain while preserving catalytic activity and hence termed as gatekeeper mutation. A similar “gatekeeper” mutation (T315I) in the BCR-ABL fusion kinase in chronic myelogenous leukemia cancer cells renders these leukemias resistant to the ABL kinase inhibitors gleevec and dasatinib, suggesting a conserved mechanism of resistance to TKIs [89]. However, the T790 M mutation may also occur prior to treatment with erlotinib or gefitinib and therefore, may contribute to primary resistance [90]. Several other EGFR mutations can also confer resistance to first generation TKIs such as D761Y and T854A [88, 91]. Interesting data also points to the possibility of additional EGFR family members such as HER2 and EGFR3 as candidates for TKI sensitivity [92, 93]. Adding to the complexity, KRAS mutations seem to grant resistance to TKIs [94]. Overall, adding erlotinib to chemotherapy does not appear to improve the survival for patients with mutations in EGFR [95, 96]. 

 In order to study the effects of the most common EGFR mutations *in vivo*, Politi et al. created doxycylcine inducible, transgenic mice that expressed an exon 19 deletion mutant or the L858R mutant in type II pneumocytes [97]. Not surprisingly, both models could recapitulate the human lung adenocarcinoma development, and were responsive to dox removal, or treatment with erlotinib. Additional studies revealed an EGFR-protein network in the plasma of these mice that included a 21-protein-network signature [98]. These networks included the TGF-*β* pathway, NF-*κ*B pathway, and the EGFR pathway. Further, the plasma EGFR mouse model network contained proteins that bind EGFR directly (Met, Cd44, Cdh1, Ndn, Sh3bgrl, and Rin1) and proteins that interact indirectly [98]. 

### 2.3. EML4-ALK

Anaplastic lymphoma kinase (ALK) is a receptor tyrosine kinase that is frequently involved in gene fusions in hematological disorders. ALK is normally not expressed in the lung [99], however fusion of ALK with upstream partner, the N-terminal echinoderm microtubule-associated protein-like 4 (EML4), have been found in 3% to 13% of NSCLC [17–20]. There are multiple EML4-ALK variants identified in lung cancer that contain variable truncations of EML4 (at exons 2, 6, 13, 14, 15, 18, and 20) fused to the kinase gene ALK beginning at exon 20 [17]. Functionally, these fusions result in protein oligomerization and constitutive activation of the kinase or elevated expression. Further, when overexpressed *in vitro*, these fusion proteins have gain of function characteristics [19, 20]. The EML4 gene is nearly always the partner gene for ALK fusions in lung cancer, although more recent studies have identified a small subset (<1%) of fusions between kinesin family member 5b and ALK (KIF5B-ALK) [100, 101], and others between TRK-fused gene (TFG) and kinesin light chain1 (KLC-1) to an even smaller percentage [102, 103].

 Similar to EGFR mutations in lung cancer, EML4-ALK mutations occur primarily in the adenocarcinoma subtype, and usually occur in never- and light-smokers [17, 18]. Further EML4-ALK mutations are mutually exclusive with KRAS or EGFR mutations. To characterize tumors formed under this oncogenic mutation* in vivo*, Soda et al. created a mouse model that expressed EML4-ALK specifically in the lung alveolar epithelial cells by using the surfactant-protein-C gene (SPC) promoter [104]. These mice developed hundreds of adenocarcinoma nodules in both lungs shortly after birth. Further, treatment with a 2,4-pyrimidinediamine derivative with a median inhibitory concentration for ALK of 10 nM and a high specificity to ALK was effective in significantly reducing tumor burden by ~30% [104]. It is important to note, however, that mice in both groups remained metastasis free, suggesting that EML4-ALK alone is insufficient to confer metastatic potential to NSCLC. 

 When seeking downstream pathways affected by EML4-ALK, and P13K, MEK/ERK pathways were not required for oncogenesis, though Hsp90 played a role [105]. Further, EML4-ALK is rapidly degraded upon exposure of cells to Hsp90 inhibitor IPI-504 [106]. This degradation leads to a potent inhibition of downstream signaling pathways and to the induction of growth arrest and apoptosis in cells carrying the EML4-ALK fusion. In addition, a xenograft model of a human NSCLC cell line containing the ALK rearrangement, tumor regression was observed at clinically relevant doses of IPI-504. Finally, cells that have been selected for resistance to ALK kinase inhibitors retain their sensitivity to IPI-504. 

 Contradictory studies have shown that forced expression of EML4-ALK-induced activation of ERK and STAT3, but not that of AKT [107]. Importantly, inhibition of ERK or STAT3 signaling resulted in substantial attenuation of the proliferation of cells expressing EML4-ALK. In addition, the specific ALK inhibitor TAE684 induced apoptosis that was accompanied both by upregulation of BIM and downregulation of Survivin. Depletion of BIM and overexpression of Survivin each inhibited TAE684-induced apoptosis, suggesting that both upregulation of BIM and downregulation of Survivin contribute to TAE684-induced apoptosis in EML4-ALK-positive lung cancer cells [107]. 

 The development of TKIs targeting the EML4-ALK fusion has been successful at targeting tumors with oncogene addiction to the mutation, that is, tumors with the gene fusion appear to be responsive to inhibitors of ALK kinase activity. The most successful compound, Crizotinib (PF-02341066), has recently been approved for treatment of NSCLC-containing ALK translocations [108]. Despite the efficacy of ALK-targeted compounds in preclinical studies, however, their efficacy is somewhat limited by the emergence of acquired drug resistance [109]. Two independent mechanisms have been suggested to contribute to this resistance. In the Crizotinib-resistant DFCI076 cell line, a unique L1152R ALK secondary mutation and concurrent coactivation of epidermal growth factor receptor (EGFR) signaling imparted resistance. In this study, a subset (3/50; 6%) of treatment naive NSCLC patients with ALK rearrangements also had concurrent EGFR activating mutations, suggesting that these mutations are not mutually exclusive and that the combination of both ALK and EGFR inhibitors may be an effective strategy for certain subgroups of NSCLC patients [109]. 

### 2.4. MET

MET is a proto-oncogene that encodes a tyrosine kinase membrane receptor (also known as hepatocyte growth factor receptor, HGFR) which can bind to the HGF ligand or scatter factor (HGF/SF). MET activation induces specific phosphorylation of several tyrosine residues, which, in turn, activates multiple downstream signaling pathways, including RAS/ERK, PI3 K/AKT, and c-SRC kinase pathways [110]. c-MET is also considered a promoter of epithelial-mesenchymal transition (EMT), due to its role in Src activation. Elevated levels of HGF and intratumoral MET expression have been associated with a more aggressive biology and a worse prognosis in NSCLC [111]. Alteration in the MET gene, including amplification, overexpression, and mutations have been described in a number of solid tumors such as papillary renal cancer, gastric cancer, and NSCLC [112]. Mutations in MET have been identified in approximately 5% of NSCLCs, mainly involving exons 2 and 14 with no clear difference in mutation frequency between histologic subtypes [113]. In comparison with renal and gastric carcinoma, mutations in the kinase domain of MET are rare in NSCLC [113, 114]. Interestingly, a mutliethnic study on 141 asian, 76 Caucasian, and 66 African American lung cancer patients revealed that the type and frequency of MET mutations were different among each group [115]. The MET mutation N375S was detected in a high proportion of East Asian samples and was correlated to incidence of squamous-cell carcinoma. This mutation also seemed to confer resistance to MET inhibition. The frequency of MET mutations was highest among male smokers. 

 In another study involving a cohort of 188 adenocarcinomas, only 3 somatic MET mutations were identified; two in exon 13 encoding the juxtamembrane domain (Arg988del and Tyr1021Asn) and one in exon 18 encoding the kinase domain (Gly1260Cys) [116]. Additionally, an intronic splice variant leading to exon 14 deletions has been reported in 2-3% of NSCLC tumors in Japanese cohorts, and this mutation led to delayed receptor downregulation and increased ligand mediated proliferation [113, 114]. 

 Of particular interest to patients harboring EGFR mutations, amplification of MET gene has been associated with secondary resistance to EGFR tyrosine kinase inhibitors through a mechanism termed as kinase switch [117, 118]. MET amplification has been reported in about 20% of tumors from patients with acquired resistance to EGFR inhibitors suggesting that a combination of MET and EGFR inhibitors might be successful in treating patients with EGFR mutations [119, 120]. However, MET amplification has been reported only in 1–7% of patients with NSCLC not treated with EGFR-specific tyrosine kinase inhibitors [114, 121]. 

### 2.5. HER2/ERBB2

 HER2/ERBB2 is another member of the ERBB family of receptor tyrosine kinases, and it can form homo- or heterodimers with other members of the family. HER2 is an unusual member of the ERBB family in that it does not interact with the EGF ligand family, but rather has an inflexible extracellular region. Once ligands engage different family members, the HER2 receptor can then heterodimerize with the ligand-bound member. Evidence suggests that HER2 acts as the preferred dimerization partner for other family members as well, and could even enhance EGFR-mediated signaling [122–124]. Following dimerization, a variety of downstream pathways can elicit activation of various kinases including the PI3 K pathway, MAPK pathway, and the JAK/STAT pathway [125, 126]. It is overexpressed in about 20% of NSCLC, though HER2 mutations occur in only 2% of NSCLC [127]. Mutations involve in frame insertions/duplication in exon 20, mostly between codon 774 and 779, resulting in the constitutive activation of the receptor [128]. Interestingly, all mutations occurred in the adenocarcinoma type cancers, and four of 5 cases were current or ex-smokers. In a contrasting study, these mutations are more prevalent in never smokers, women and asian patients and more frequent in adenocarcinoma than in other histological types of NSCLC [127, 129]. In another study of 504 Japanese lung cancer patients, HER2 mutations were identified in 13 of 504 cases (2.6%) [130]. The subgroup of nonsmokers with adenocarcinoma or adeno-squamous-cell carcinoma without EGFR mutations harbor a frequency of HER2 mutations of 14.1% (11/78). HER2 mutations are not present in tumors harboring EGFR or KRAS mutations. 

 Given that HER2 mutations and amplification is observed in a variety of human cancers, targeting HER2 has been an effective modality for inhibiting tumor growth and progression. A monoclonal antibody that targets HER2, Trastuzumab (Herceptin) can induce downregulation of HER2 and cell-cycle inhibition [131]. Further, the reversible small molecule inhibitor of both EGFR and HER2, Lapatinib (GW572016), has also shown modest efficacy in downregulation of Src and AKT signaling [131, 132]. Unfortunately the use of these single agents in phase II clinical trials was disappointing [133].

### 2.6. B-RAF

Nearly a decade after the discovery of RAS as a human oncogene, the first critical effector protein was identified—RAF-1 serine/threonine kinase [134, 135]. This protein, along with its two closely related family members A-Raf and B-Raf, are responsible for triggering the mitogen-activated protein kinase (MAPK) pathway [136]. Recent studies have revealed that 60% of melanomas harbor activating mutations in the B-RAF kinase gene, and in some colon, thyroid, and lung cancers as well [137]. In total, B-RAF mutations occur in approximately 7% of all human cancers [138]. The most common B-RAF mutation, being most frequent in melanoma at 80%, is the glutamic acid for valine substitution at position 600 (exon 15), which produces a 500-fold activated protein that signals to MEK-ERK constitutively, conferring the cell with increased survival and proliferation [138, 139]. These mutations in some cases cause constitutive heterodimerization with C-RAF [140]. 

 In contrast to the most common B-RAF mutation, NSCLC have mostly non-V600E mutations, including D594G and L596R mutation in the kinase domain, and G465V or G468A mutations in the G-loop of the activation domain [138, 141–143]. Importantly, B-RAF missense mutations were observed in 4 out of 35 lung adenocarcinoma cell lines tested (11%), but not in 14 primary lung cancers analyzed [138]. More recently, however, one study showed that out of 697 patients with lung adenocarcinoma, all patients harboring B-RAF mutations (18 patients; 2.6%) were former or current smokers (*P* < 0.001) [143]. 

 The heterogeneity of B-RAF mutations observed in lung cancer makes the use of PLX4032 (the promising small molecule B-RAF kinase inhibitor designed to target the V600E mutation) less desirable [144]. In addition to the complexities associated with the precise mutation-specific actions of this new drug, resistance can develop to the inhibitor. Various studies have identified mechanisms for acquired resistance in melanoma to mutations in upstream regulators of the ERK pathway including NRAS, MAP3 K8, PDGF, and IGF-1 receptor tyrosine kinases [145–147]. These changes can induce cell proliferation irrespective of mutant B-RAF. 

### 2.7. MEK-1

MEK1 (also known as MAP2 K1) is one of the pivotal downstream effectors of RAS-signaling cascades in NSCLC. Mek1 is a serine-threonine kinase that primarily activates ERK1 and ERK2 downstream of RAF family members [148]. In a cohort of lung adenocarcinoma, 2 out of 207 (~1%) primary lung tumors had somatic activating mutations in exon 2 of MEK1, a K57N mutation in the nonkinase portion of protein [63]. In addition, this residue is highly conserved from Arabidopsis to humans [63]. Further proving that mutations in the RAS-RAF-MAPK pathway often have one hit per tumor, these tumors had no other known mutations in genes often mutated in lung cancer, such as EGFR, KRAS, HER2, or PIK3CA, or BRAF. Further, expression of mutant MEK1 led to the constitutive activation of ERK1 and ERK2 in 293T cells. Treatment of 293T cells with the small-molecule MEK inhibitor AZD6244 completely abrogated downstream phosphorylation of ERK—suggesting that this compound might be efficacious for patients harboring this rare mutation. Sasaki et al. have also identified the MEK1 K57N mutation in 1 out of 241 human lung adenocarcinoma samples (0.4%) [149]. 

 Whether the MEK mutation in NSCLC is a driver mutation is still not determined, mostly because of the rare case of mutation in humans. In an orthotopic mouse model with NSCLC cell lines, MEK inhibition could significantly decrease angiogenesis, VEGF expression, and sequential signaling [150]. Further interruption of both STAT3-survivin and ERK-BIM pathways was critical for induction of apoptosis in NSCLC harbouring EML4-ALK—this was accomplished using ALK and MEK inhibitors in EML4-ALK-positive NSCLC patients for whom ALK inhibitors alone are ineffective [151].

### 2.8. PIK3CA and AKT

 PI3Ks are a family of intracellular, heterodimeric lipid kinases that phosphorylate the 3′ hydroxyl group of phosphatidylinositols and phosphoinositides. PI3K pathway regulates diverse cellular processes including cell proliferation, survival, metabolism, apoptosis, and cell migration [152]. Among the four different isoforms of the p110 catalytic subunit of PI3K, *PIK3CA,* the gene encoding the p110*α* catalytic subunit, is the only gene frequently mutated in cancer; these mutations occur in the helical or kinase domains of the catalytic subunit [152]. Along with KRAS, it is believed that PI3K mutations are the second most common mutations in oncogenes in cancer. However, mutations of this gene have been identified in 30% of glioblastomas and gastric cancers, but are much less frequent in lung cancers [153]. In fact, only 2% of NSCLC cases show mutations in PIK3CA where these mutations most frequently affect residues Glu542 and Glu545 in exon 9 encoding the catalytic domain. In addition to mutations, this study also identified PIK3A copy number gains, which were more frequent in squamous-cell carcinoma (33.1%) than in adenocarcinoma (6.2%) or SCLC lines (4.7%), making this aberration one of the few more prevalent in the squamous histological subtype [154]. Previous studies have shown that a region of chromosome 3q (3q25–27), where PIK3CA (3q26) is located, is frequently amplified in lung cancers, especially squamous-cell carcinomas. In another study, PIK3CA amplification was significantly associated with smoking history and histological type, which was more frequent in smokers compared to never smokers, and in squamous-cell carcinoma compared to adenocarcinoma [155].

 Although the exact mechanism of tumorigenesis from PIK3CA mutations is unclear, PIK3CA mutations lead to enhanced PI3 K enzymatic activity *in vitro* and growth-factor-independent activation of Akt/Protein kinase B signaling pathways resulting in oncogenic transformation. In addition to mutations, PIK3CA is frequently amplified in NSCLC, particularly in men, smokers, and also in squamous-cell carcinoma. The primary downstream mediator of PIK3CA, AKT, or protein kinase B is a serine threonine kinase that is activated by PI3 Kinase and represents a key node in the PI3K pathway. Interestingly, a major recurrent mutation (E17K) in the AKT1 gene has been identified in various cancers including breast, ovarian, and colon cancers [156]. This mutation occurs in the AKT1 pleckstrin homolog domain and alters the phosphoinositide-binding pocket, and leads to PI3K-independent AKT activation. Although, AKT1 mutations are rare in lung cancer (1.9%), the oncogenic properties of E17K mutaions might also contribute to the development of a fraction of lung carcinoma with squamous histotype (5.5%) [157]. 

### 2.9. TTF1 (NKX2.1 or TITF1)

 Thyroid Transcription Factor 1, TTF1, or TITF1, also known as NK2 homeobox 1 (NKX2.1), is a transcription factor essential for the development of normal lung airways, thyroid, and brain (Boggaram, 2009 #356). Particularly in the lung, NKX2.1 participates in differentiation of cells into lung branches, and its expression is restricted to certain cells assigned to stringently maintain the lung architecture. Interestingly, NKX2.1 expression can be detected in a wider range of NSCLCs (around 50%), which suggests that NKX2.1 might contribute to the development of these cancers [158–161]. Further highlighting a role for NKX2.1 in the lung development, several mouse models have provided evidence: knockout mice have defects in branching morphogenesis, and results in neonatal death. Although mutations that prevent NKX2.1 phosphorylation result in relatively normal morphogenesis, but exhibit lethal functional defects including abnormalities in acinar tubules and pulmonary hypoplasia indicating defects in lung morphogenesis later in development [162]. In a transgenic mouse model, increased expression of TTF1 in respiratory epithelial cells inhibited alviolarization and caused pulmonary inflammation demonstrating that precise regulation of TTF1 is critical for homeostasis in the postnatal lung. Modest overexpression of TTF1 caused type II cell hyperplasia and increased the cellular content of pulmonary surfactant protein B (SP-B). In contrast, higher expression levels of TTF1 disrupted alveolar septation, causing emphysema. In mice with the highest transgene expression, TTF1 caused severe inflammation, pulmonary fibrosis, respiratory failure, and death, associated with eosinophil infiltration, and increased expression of eotaxin and IL-6 [163].

 In human lung, NKX2.1 haploinsuffficiency causes respiratory dysfunction, abnormal airway and alveolar morphogenesis, and abnormal surfactant protein expression and infections [164]. Amplification of the 14q13.3 locus harboring NKX2.1 gene is observed in 7–15% of lung cancer cases [165, 166] and 33% of lung cancer cell lines. Knockdown of TTF1 in lung cancer cell lines with amplification led to reduced cell proliferation, manifested by both decreased cell-cycle progression and increased apoptosis indicating that TTF1 is a lineage-specific oncogene in lung cancer [158]. Further, an increase in the gene dosage of TTF1 in 214 patients with NSCLC (including 174 adenocarcinomas) showed, a higher frequency of increased gene copies at metastatic sites than at primary sites suggesting that sustained TTF1 expression may be crucial for survival of a subset of adenocarcinomas [161]. Thus, TTF1 is essential for the development of the peripheral airways and is a lineage-specific marker for tumors developing from the terminal respiratory unit, that is, peripheral ADCs. Several lines of evidence suggest that Nkx 2.1 is an adinocarcinoma lineage-specific target gene [161, 167] and it is not expressed in squamous-cell carcinoma (SCC) [168, 169]. A recent study indicated that 14q amplification does occur in SCC, however, FOXA1 gene, located only 1Mbp downstream of NKX2.1 might be the target gene in SCC [170]. Genome-wide analyses of NKX 2.1 binding to transcriptional target genes uncovered differential Nkx2.1-regulated networks in early and late lung development and a direct function in regulation of cell cycle by controlling the expression of proliferation-related genes such as E2F3, Cyclin B1, Cyclin B2, and c-Met [171].

 Although several studies demonstrated NKX2.1 to be a lineage-specific oncogene and its expression was found to be crucial for the survival of a subset of adenocarcinomas [161, 167], a recent mouse model links NKX2.1 downregulation to a loss in differentiation, enhanced tumor-seeding ability, and increased metastatic proclivity [172]. Thus, the oncogenic and tumor suppressor functions of Nkx2.1 within the same tumor type support its role as a dual-function lineage factor [172]. Hence it is not surprising that numerous studies assessing the prognostic role of Nkx2.1 in lung cancer reported inconsistent results [159, 161, 167, 173–176].

### 2.10. ROS

The transmembrane proto-oncogene receptor tyrosine kinase (RTK) ROS is receptor kinase of insulin receptor family that is aberrantly expressed in neoplasms of the central nervous system. Chromosomal rearrangements involving the ROS1 gene were originally described in gioblastomas, where ROS1 (chromosome 6q22) is fused to the FIG (Fused in Glioblastoma) gene (chromosome 6q22 immediately adjacent to ROS1) [177]. In transgenic mouse models, FIG-ROS expression led to the formation of glioblastomas and that formation of these tumors were greatly accelerated in the absence of tumor suppressor genes *p16Ink4a *and* p19Arf* [178]. ROS1 fusions were identified as potential driver mutations in an NSCLC cell line (HCC78; SLC34A2-ROS1) and an NSCLC-patient sample (CD74-ROS1) in a large-scale survey of tyrosine kinase activity in lung cancer using phosphoproteomic approaches [103]. Recently, ROS1 rearrangements were identified in 1.7% (18 out of 1073) patients with NSCLC using fluorescence in situ hybridization while 2.9% were ALK rearranged [179]. Patients with ROS1 rearrangements were significantly younger and more likely to be never smokers and all of the ROS1-positive tumors were adenocarcinomas with a tendency toward higher grade. Interestingly, these clinical features were similar to those associated with EGFR mutations and ALK rearrangements [127, 180] and preclinical studies using a kinase inhibitor TAE684, effectively inhibited the growth of the HCC78 cell line harboring ROS1 translocation [181]. In addition, ALK/MET inhibitor crizotinib also inhibited growth of HCC78- and ROS1-positive tumors suggesting that lung cancer patients with ROS1 rearrangement could benefit from targeted therapy using crizotinib [179].

### 2.11. RET

The *RET* gene (rearranged during transfection) on chromosome 10q11.2 encodes a receptor tyrosine kinase that normally plays a crucial part in neural crest development [182]. More than 20 years ago, RET gene was shown to be associated with papillary thyroid carcinoma (PTC) through chromosomal rearrangements (RET/PTC) [183]. Somatic and germline point mutations occur in sporadic and familial medullary thyroid cancers, respectively. RET fusions (involving *CCDC6*, *PRKAR1A*, *NCOA4* (*ELE1*), *GOLGA5*, *TRIM24 (HTIF1)*, *TRIM33* (*RFG7*), and *KTN1* and *ERC1* (*ELKS*)) are found in papillary thyroid cancers [184, 185]. Currently, an inhibitor specific for only RET is not available, but trials of kinase inhibitors with anti-RET activity have been conducted in thyroid cancer, leading to U.S. Food and Drug Administration (FDA) approval of one (vandetanib) for the treatment of adults with metastatic hereditary medullary thyroid cancers [186]. Although RET fusions have not previously been described in lung cancer, a recent study identified in-frame fusion transcripts of *KIF5B* (the kinesin family 5B gene) and the *RET* oncogene, which are present in 1-2% of lung adenocarcinomas (LADCs) from people from Japan and the United States, using whole-transcriptome sequencing [21]. The *KIF5B-RET* fusion led to aberrant activation of RET kinase and is considered to be a new driver mutation of lung adenocarcinoma because it segregates from mutations or fusions in *EGFR*, *KRAS*, *HER2,* and *ALK.* Additionally, RET tyrosine kinase inhibitor, vandetanib, suppresses the fusion-induced anchorage-independent growth activity of NIH3T3 cells [21]. In another study, combined analysis of massively parallel whole-genome and transcriptome sequencing for cancer and paired normal tissue of a 33-year-old lung adenocarcinoma patient, who is a never-smoker and has no familial cancer history revealed the presence of the fusion gene between KIF5B and the RET proto-oncogene caused by a pericentric inversion of 10p11.22-q11.21 [22]. This fusion gene overexpressed chimeric RET receptor tyrosine kinase, which could spontaneously induce cellular transformation. Further, they identified the KIF5B-RET fusion in two more cases out of 20 primary lung adenocarcinomas in the replication study demonstrating that a subset of NSCLCs could be caused by a fusion of KIF5B and RET, and suggesting the chimeric oncogene as a promising molecular target for the personalized diagnosis and treatment of lung cancer.

 In a similar study, using a next-generation sequencing assay targeting 145 cancer-relevant genes in 24 non-small-cell lung cancer formalin-fixed paraffin-embedded tissue specimens identified *KIF5B-RET* fusion in lung adenocarcinoma. Further screening of 561 lung adenocarcinomas identified 11 additional tumors with *KIF5B-RET* gene fusions [23]. 

 Each of these studies discovered RET fusions involving the *KIF5B* (kinesin family member 5B) gene, which encodes a coiled coil domain thought to mediate dimerization^.^ Under normal circumstances, KIF5B is part of a motor protein complex that is responsible for organelle trafficking [187]. Collectively, these studies identified a total of seven KIF5B-RET fusion variants, in all seven variants, as with other kinase fusions, the breakpoint left the RET kinase domain portion intact. The fusions occurred predominantly in adenocarcinomas from never smokers and were mutually exclusive of mutations in *EGFR*, *KRAS,* and *ALK.*

## 3. Inactivation of Tumor Suppression Pathways

### 3.1. TP53 Mutations

 Alteration in the TP53 gene is one of the most significant events in lung cancers and plays an important role in the tumorigenesis of lung epithelial cells. Approximately 40–60% of NSCLCs and 70% of SCLCs have mutations in the tumor suppressor gene TP53, regardless of their EGFR or KRAS mutation status [188, 189]. Somatic TP53 missense mutations are found in approximately 50% of human cancers, and inactivating mutations in the TP53 gene are the most common genetic events in human cancers affecting a specific gene, with the vast majority arising from a single-point mutation in the segment encoding the DNA-binding domain of TP53 [190, 191]. The inactivating mutations render the mutant TP53 protein unable to carry out its normal functions, that is, transcriptional transactivation of downstream target genes that regulate cell cycle and apoptosis [192]. Several recent studies indicate that the common types of cancer-associated TP53 mutations also endow the mutant protein with new activities, so-called “gain-of-function” (GOF) activities, which can contribute actively to various stages of tumor progression, including distant metastases, and to increased resistance to anticancer treatments. GOF activities of mutant TP53 are exerted by aberrant protein interaction or gene regulation, such as MAPKK3, inhibitor of DNA-binding 4 (ID4), polo-like kinase 2 (Plk2), promyelocytic leukemia protein (PML), and prolyl isomerase Pin1 [193–195]. Although the occurrence of TP53 mutations is not limited to a few particular sequences or codons along this gene, most mutations cluster in the TP53 DNA-binding domain [196]. Most TP53 missense mutations lead to the synthesis of a stable protein, which lacks its specific DNA-binding and transactivation function and accumulates in the nucleus of cells. These mutant accumulated proteins are retained in distant metastasis and also shown to be capable of cooperating with oncogenes for cellular transformation [197]. It is reported that five of the six most prominent mutation hotspots in the TP53 gene are represented by G to T mutations at codons containing methylated CpG sequences, including codons 157, 158, 245, 248, and 273 [198]. The understanding of the tumor-specific mutational spectra of the TP53 gene is quite important for the understanding of TP53-associated carcinogenesis. Analysis of the spectrum of TP53 mutations in human cancer demonstrates a link between exposure to various types of carcinogens and the development of specific cancers [199]. For example, these mutations are less common in the lung cancers of never smokers than in tobacco-associated lung cancers [199]. Moreover, the types and spectra of TP53 mutations differ significantly according to the smoking status of the patient [200].

 The frequency of G-to-T transversions is higher in smokers, whereas that of G-to-A transitions is higher in never smokers [113, 200]. The G-to-T transversions usually occur at bases that serve as binding sites for adducts of polycyclic aromatic hydrocarbons [201]. Another study indicated that the G-to-T : G-to-A ratio was 1.5 in women smokers and 0.23 in women never smokers [202]. Moreover, mutations at codons 157, 158, 245, and 248 (“warm spots”) of TP53 gene were less frequent in never smokers [201, 203]. Further studies indicated that the TP53 mutations in women never smokers with adenocarcinoma were predominantly transitions (83%); however, in smokers, the mutations were predominantly transversions (60%) and deletions (20%) [204]. 

 The frequent detection of loss of heterozygosity (LOH) in lung cancer cell lines and tumor samples at the location of the TP53 gene on chromosome 17p13 suggested that this gene was likely to be involved in the pathogenesis of lung cancer, and genetic abnormality of the TP53 in lung cancers has been shown to be associated with a poorer survival prognosis and increased cellular resistance to therapy [205]. The highest frequency of TP53 alterations is found in SCLC specimens. On the other hand, the frequency of TP53 mutations is the highest in squamous-cell carcinomas and lower in adenocarcinomas among NSCLC-tumor samples [206]. It has been reported that somatic mutations and increased expression of TP53 were frequently found in ~23% and ~65% of NSCLC, respectively [207]. TP53 mutations are found in tumors both with and without allele loss at 17p13 and are mostly located within the DNA-binding domain of TP53 [208]. Because coding mutations of TP53 occur relatively early in the development of lung cancer and are potentially required for maintaining the malignant phenotype, the acquired TP53 mutations are preserved during tumor progression and metastatic spread [209]. It has been reported that the incidence of TP53 mutations in primary tumors and metastatic lymph nodes was 23.2% and 21.4%, respectively, and the TP53 gene status in primary tumors and metastatic lymph nodes showed 92.9% concordance among 56 patients with NSCLC who had undergone surgical resection, which explained the fact that TP53 mutations usually precede lymph node metastasis [210]. Most TP53 mutations occur before the tumor metastasizes. They are then preserved through subsequent stages of tumor development; as a result, no selection against TP53 mutations occurs during metastasis.

 The role of mutant TP53 in the prognosis of lung cancer is a matter of controversy; some reports suggest a negative prognostic effect while others report a positive or no effect [211]. A meta analysis of 43 published reports concluded that TP53 mutations as determined by IHC and mutational analysis were a significant marker of poor prognosis in patients with pulmonary adenocarcinoma [212], and this observation was later confirmed by other groups [213–215]. Several studies suggest that TP53 mutations confer chemoresistance to lung cancer cells *in vivo* and *in vitro* [205], supporting its association with poor prognosis.

### 3.2. PTEN Mutations

 Phosphatase and tensin homolog deleted on chromosome 10 (PTEN) is a tumor suppressor gene encoding a 403 amino-acid-dual-specificity lipid and protein phosphatase [216]. PTEN negatively regulates the phosphatidylinositol 3-kinase (PI3K) signaling pathway by dephosphorylating PI-(3,4,5)-triphosphate, which mediates activation of AKT. This results in inhibition of PI3 K-AKT-mTOR pathway leading to G1 cell cycle arrest and apoptosis. In addition, PTEN inhibits cell migration and spreading through its regulation of focal adhesion kinase as well as regulates TP53 protein levels and activity [217–219]. The PI3 K-PTEN signaling network functions as a crucial regulator of cell survival decisions [220]. When PTEN is deleted, mutated, or inactivated, activation of PI3 K effectors especially, AKT/Protein kinase B can occur in the absence of any exogenous stimulus resulting in tumorigenesis. 

 Frequent somatic mutations in the PTEN gene have been reported in a variety of sporadic tumors, including endometrial cancers and prostate cancers [221, 222]. In contrast to these tumors, PTEN mutations have been reported to occur rarely in non-small-cell lung cancer (NSCLC) [79] probably due to the small number of samples included in these studies. However, loss of heterozygosity of PTEN has been reported to occur frequently (~50%) in NSCLC [223]. A recent study tried to investigate the relationship between PTEN mutations and EGFR, KRAS, and TP53 mutations in 176 surgically resected NSCLCs. PTEN mutations were present in 8 (4.5%) of the 176 tumors, and one case concurrently had an EGFR mutation and 4 cases had TP53 mutations. However, PTEN mutations were not found in the tumors with KRAS mutation. PTEN mutations were only found in ever smokers and were significantly more frequent in squamous-cell carcinoma than in adenocarcinoma [224]. These findings indicate that PTEN mutations are relatively common in NSCLC, and thus analysis of PTEN mutations may facilitate a comprehensive understanding of the genetic alterations related to the EGFR signaling pathway.

### 3.3. LKB1

 Germline mutations in LKB1, also called STK11 (serine-threonine kinase 11), cause the autosomal dominant Peutz-Jeghers syndrome (PJS) [225, 226], which bestows an increased risk of developing a wide range of cancers, including lung cancer [227]. In humans, LKB1 is located on the short arm of chromosome 19, and encodes a CAMK-family serine threonine kinase. Functionally, LKB1 can phosphorylate a variety of downstream targets in the cytoplasm, although the best studied is AMP-activated protein kinase (AMPK), a key regulator of cellular metabolism and glucose uptake [228]. LKB1 is also known as a tumor suppressor gene, since the deletion of this gene is observed in various cancers. LKB1 has varied mechanisms of action—through the inhibition of mammalian target of rapamycin (mTOR), regulation of the cell cycle and proliferation, and even regulation of metastasis [229–231]. Aside from the somatic LKB1 deletions observed in somatic tumors, mutations by other means, such as frameshift, nonsense, missense, or large intragenic deletions, which generate truncated proteins, are also observed in lung cancer [232]. These mutations are far too heterogeneous to characterize in this paper, occuring in exons 1–8 [232].

 To highlight a role for LKB1 in mouse models of NSCLC, Kwok-Kin Wong's group created a mouse that harbored the KRasG12D mutation and homozygous inactivation of LKB1 [233]. In these mice, LKB1-deficient tumors demonstrated shorter tumor latency than mice with KRAS mutation alone, and mimicked the human spectrum of lung pathologies, having adeno-, squamous-, and large-cell carcinoma in addition to more frequent metastasis compared to tumors with TP53 mutation or Ink4a/Arf. Similar to other studies, they found that 34% of 144 human adenocarcinoma samples and 19% of squamous-cell carcinomas had inactivation of LKB1 [233, 234]. Further, gene expression profiles on human lung cancer cell lines and mouse lung tumors identified a variety of downstream genes implicated in metastasis to be upregulated following LKB1 loss, including NEDD9, VEGFC, and CD24. Whether these genes are also affected in human tissue samples remains to be demonstrated. 

 LKB1 might also play a role in the epithelial to mesenchymal transition [229]. When genomic and proteomic analysis were compared in a cross-species comparison of mouse and human samples, there was a similar pattern of expression during progression of LKB1-deficient tumors to metastases—faithfully recapitulating advanced incurable disease in human primary NSCLC. In addition, LKB1-deficient tumors had a provocative gene signature, which included up-regulation of SRC, FAK, TGF-*β*, E2F1, and stem-cell markers OCT4 and TCF3.

### 3.4. p16^INK4A^

The cyclin-dependent kinase (CDK) inhibitor p16 (p16^INK4A^/CDKN2/MTS1) was the first of four INK4 genes discovered, and is a crucial component for stringent regulation of the cell cycle [235]. It functions to inhibit cyclin-D dependent phosphorylation of pRB, and its related family members p130 and p107, by replacing cyclin D in cdk4/6-cyclin D complexes [235]. This inhibition of pRb phosphorylation keeps pRB active on E2F-target gene promoters that are required for entry into S-phase, hence sequestering E2F transcriptional activity, and inhibiting progression through the G1/S checkpoint [236]. Genetic alterations of p16^INK4A^ thusly lead to unrestricted ectopic cell proliferation through the loss of G1 arrest control. Since the loss of this critical gene occurs in several cancers including NSCLC, p16^INK4A^ is recognized as a bona fide tumor suppressor gene [237–239]. 

In human NSCLCs, aberrations in p16^INK4A^ occur with a rather high frequency (~17–58%) and is usually through homozygous deletions, though inactivating point mutations, and methylation at the 5′ CpG islands also silence p16^INK4a^ activity [113, 240–242]. Other studies have shown that IHC is a straightforward method for detection of p16 inactivation as well [243]. To determine the overall incidence of p16 mutations in biopsied NSCLC samples, Brambilla et al. examined a cohort of 168 samples using IHC. Surprisingly, 98 out of 168 (58%) had lost immunoreactivity to p16 antibodies [244]. However in univariate analysis, p16 negative cases had longer survival than p16 positive cases (*P* = 0.02), suggesting that p16 loss may not result in an unfavorable role for tumor progression and patient outcome. In one contrasting study, 244 human-NSCLC-tumor samples were analyzed by fluorescence-based, real-time methylation-specific PCR to examine the prognostic relevance of p16 DNA promoter methylation [245]. These data demonstrated that patients with hypermethylation of the p16 promoter had a negative correlation with survival (*P* = 0.0002), suggesting that deletion of this cdk-inhibitor contributed to poor prognosis. 

## 4. Conclusions

Characterization of genomic aberrations including copy number changes, nucleotide sequence changes, chromosomal rearrangements and epigenetic alterations, and elucidation of their role in carcinogenesis have provided a deep insight into the molecular events that facilitate the genesis and progression of non-small cell lung cancer. It is clear that multiple pathways, including those that promote the growth of tumors as well as those which suppress tumor growth are altered in human NSCLC. It is clear that targeting the activating mutations and their downstream biochemical pathways is more pliable and practical in developing novel therapeutics. At the same time, attempts to target signaling pathways that inhibit the function of tumor suppressive pathways are also gaining attention. Development of agents like nutlin that restores the level of TP53 is a prime example. It may be concluded that the new data derived from genomewide screening efforts, deep sequencing as well as large-scale gene expression profiling will provide additional leads into potential molecular targets that can be manipulated for therapeutic purposes. Success of such efforts will lead to improving the prognosis and quality of life of thousands of NSCLC patients around the world. 
